# Are subjective cognitive complaints associated with executive functions and mental health of older adults?

**DOI:** 10.1007/s10339-022-01089-y

**Published:** 2022-04-05

**Authors:** Maria Chiara Fastame

**Affiliations:** Department of Pedagogy, Psychology, Philosophy, University of Cagliari, Via Is Mirrionis 1, 09123 Cagliari, Italy

**Keywords:** Subjective memory complaints, Aging, Executive functions, Cognitive failures questionnaire, Older adults, Optimism

## Abstract

Subjective cognitive complaints are used to detect detrimental age-related variations in cognitive efficiency before cognitive decline occurs in late adulthood. Despite this, there is controversial evidence on the relationship between the aforementioned metacognitive measure and the actual cognitive efficiency of older individuals. Instead, subjective cognitive complaints seem to be related to perceived mental health. This study aimed to investigate the nature of the relationships between subjective cognitive failures, mental health, and executive functioning. An additional goal was to examine whether there were significant differences in perceived mental health and executive functions efficiency by comparing older people who exhibited fewer subjective cognitive complaints with a group who reported more cognitive complaints. Eighty-nine community-dwellers (*M*_age_ = 78.6 years, SD = 6.5 years; age range = 66–95 years), 42 males and 47 females, were recruited and completed a battery of tools assessing cognitive failures, depressive symptoms, psychological well-being, optimism, global cognitive functioning, vocabulary, and several executive functions. Significant relationships were only found between self-reported cognitive failures, depressive symptoms, optimism, and psychological well-being. Moreover, participants who reported more cognitive failures also exhibited less optimism and psychological well-being and showed more depressive symptoms than older respondents who exhibited fewer cognitive complaints. Finally, no differences in the measures of executive functioning were found between groups exhibiting low vs. high levels of subjective cognitive complaints. In conclusion, the concurrent objective assessment of cognitive functioning and self-reported evaluation of cognitive processes and mental health of older people should be encouraged, to detect possible threats to their well-being.

## Introduction

In clinical practice, the detection of cognitive impairment is a complex process that requires examining individual characteristics, the efficiency of distinct mental functions, and the connections among different cognitive domains, to highlight how they are related to healthy and pathological aging (Tosi et al. 2020). From an applied viewpoint, this implies not only the objective assessment of a set of cognitive processes (e.g., executive functions, vocabulary, non-verbal reasoning, text comprehension) but also the self-rating of one’s cognitive resources. The main purpose of this paper is to shed light on the link between subjective cognitive complaints, objectively assessed cognitive efficiency, and self-reported psychological well-being in late adulthood. Subjective cognitive complaints refer to the self-reported assessment of one’s cognitive functioning that reflects the perceived efficiency of memory processes and cognitive skills implicated in daily life (e.g., failing to notice certain evident elements present in the environment, postponing doing something for days, and dropping things accidentally). Therefore, subjective cognitive complaints are a measure of metacognitive functioning since the individual reflects on his/her mental processes and how he/she can monitor and control them (Mecacci and Righi 2006).

It is well-established that in late adulthood, subjective cognitive complaints expressed by cognitively healthy older individuals may be useful in detecting early variations in cognitive ability prior to the clinical and objective diagnosis of Alzheimer’s Disease in combination with amyloid deposition (Sperling et al. 2011). Thus, as pointed out by a research trend (Jack et al. 2018; Mendonça et al. 2016), subjective cognitive complaints may be carefully considered as self-reported behavioral pre-clinical markers of cognitive impairment. Moreover, there is evidence that the hippocampus plays a crucial role in information encoding (Kim 2011) and memory processing (Squire 1992) and that patients who exhibit early stages of Alzheimer’s Disease in combination with amyloid deposition also show decreased hippocampal volume (Petersen et al. 2016). Following these findings, a recent longitudinal study documented that in typical normal aging, decreased hippocampal activation predicted increased cognitive complaints and higher amyloid pathology, especially in individuals beginning to enter late adulthood (i.e., below 69 years old) (Chen et al. 2021). Furthermore, it was also shown that increased subjective memory concerns were associated with the decline in objectively assessed memory processes (Chen et al. 2021).

Regarding the predictive role of subjective cognitive complaints on actual memory decline in late adulthood, the experimental evidence is controversial. For instance, Jungwirth et al. (2004) found no significant associations between subjective memory complaints and objectively assessed memory skills in a sample of cognitively healthy older participants. Consistently, in a longitudinal study, no significant relationships were found between actual memory changes and initial memory complaints and variations in memory complaints expressed over the course of six years by a sample composed of typically developing older individuals (Pearman et al. 2014). Furthermore, Shakeel and Goghari (2017) did not find significant associations between subjective cognitive complaints assessed through the Cognitive Failures Questionnaire (CFQ) (Broadbent et al. 1982; see Method section), fluid intelligence, and several measures of executive functions (i.e., cognitive flexibility, planning, and non-verbal fluency) evaluated in a sample of participants over 65 years old. In contrast, Fastame et al. (2014b) found that community-dwelling older participants with normal cognition who reported fewer cognitive lapses and errors assessed through the CFQ (Broadbent et al. 1982), also exhibited a better passive (i.e., temporary information maintenance) and active (i.e., information processing) verbal and visuo-spatial working memory. Consistent with this, Hohman et al. (2011) documented that the CFQ (Broadbent et al. 1982) scores of cognitively healthy older individuals predicted longitudinal changes in immediate recall but not in executive functions (e.g., phonological and categorical verbal fluency) over a period of 11 years. Moreover, the authors showed that high levels of cognitive complaints were associated with increased bilateral insula and cerebellum activity and with the activation of the right inferior parietal cortex, left lingual gyrus, and right fusiform gyrus. In addition, another study (Jonker et al. 1996) showed significant positive relationships between subjective cognitive complaints and worse delayed recall, concentration, and categorical verbal fluency in late adulthood. Overall, these findings do not allow us to track univocal conclusions about the relationship between subjective cognitive concerns, memory, and executive function processes in healthy aging.

Furthermore, little is known about the role played by other psychological factors in predicting the occurrence of subjective cognitive complaints. Concerning this issue, recently, Zullo et al. (2021) argued that cognitive complaints in older individuals are predicted by higher neuroticism and depressive symptoms and are associated with lower extraversion and worse categorical verbal fluency skills. A related research trend showed that global psychological well-being in healthy aging was predicted by self-reported depressive symptoms and two metacognitive measures, including CFQ score (Fastame et al. 2014a). Consistent with this, there is also evidence (Fastame and Penna 2014) that 75–99-year-old individuals living in the Sardinian Blue Zone (i.e., an area located in the central-eastern mountainous region of the Mediterranean island characterized by higher levels of longevity and successful aging) self-reported better psychological well-being (i.e., personal satisfaction and emotional competences) and exhibited fewer depressive symptoms and lower CFQ (Broadbent et al. 1982) scores than older individuals living in a rural area located in northern Italy. Finally, extending this evidence, a recent longitudinal study (Fastame et al. 2021) documented the greater psychological well-being of successful older individuals living in the Sardinian Blue Zone over two years, whereas their CFQ (Broadbent et al. 1982) scores remained stable at follow-up. However, a methodological limitation of the studies conducted in the Sardinian Blue Zone is that psychological well-being was assessed using a questionnaire that has only been validated for the Italian geriatric population and is not currently designed to evaluate subjective mental health elsewhere. Therefore, at present, the possibility of replicating the above-mentioned investigations in other cultural settings to examine the relationship between mental health measures and subjective cognitive complaints is limited. Moreover, to date, no studies have been conducted to investigate the nature of the relationships between subjective cognitive complaints, psychological well-being, and optimism in late adulthood. In order to promote mental health in the last decades of life, exploring these relationships is crucial, as there is evidence that optimism may have a significant impact on mental well-being, and a more marked optimistic attitude is associated with a higher quality of life and better adaptive behaviors for coping with stressful events (Conversano et al. 2010).

This study primarily aimed to evaluate whether subjective cognitive complaints in late adulthood were associated with perceived mental health and objectively assessed executive functions. To pursue this goal, two groups exhibiting high vs. low levels of cognitive complaints were established. Therefore, measures of perceived mental health and executive functions of older individuals reporting low and high CFQ scores (Broadbent et al. 1982) were examined. Specifically, the current study intended to investigate the following: (1) the nature of the relationships between the CFQ (Broadbent et al. 1982) scores and several self-reported psychological well-being measures (i.e., including optimism) and objectively assessed executive functions, respectively; (2) whether older individuals reporting higher CFQ (Broadbent et al. 1982) scores also reported lower mental health indices and showed worse measures of executive functioning. Overall, following Zullo et al. (2021), the current study intended to provide a better understanding of the link between several psychological factors (i.e., the efficiency of executive functions and mental health) and a well-known self-reported metacognitive functioning measure that assesses perception, motor, and memory errors in everyday life, namely the CFQ (Broadbent et al. 1982). That is, by embracing an applied perspective, the emerging evidence could provide useful information for the implementation of preventive and empowering interventions aimed at improving the quality of life in the last decades of life.

To pursue the second goal, an approach based on the study of individual differences was adopted, that is, older participants who exhibited very high levels of subjective cognitive complaints (i.e., Complainers) and peers who self-reported very few cognitive errors and lapses (i.e., Non-Complainers) were selected and compared on a series of measures of self-reported mental health and objectively assessed executive functions. In addition, to facilitate the replication of this study in socio-cultural contexts other than the Italian one, in the current study, mental health was assessed through some well-known tools widely used internationally (see the Materials section for a more detailed description of the inventories).

Thus, keeping in mind the exploratory nature of this study, in line with previous evidence (Fastame et al. 2014a), significant associations were hypothesized between the CFQ score (Broadbent et al. 1982) and the measures of psychological well-being and depressive symptoms, respectively. Moreover, Complainers were expected to self-report more depressive symptoms and less psychological well-being than participants reporting fewer cognitive failures (Fastame and Penna 2014). Furthermore, due to the lack of previous evidence, no a priori hypotheses were proposed on the relationship between subjective cognitive complaints and self-reported optimism in late adulthood. Finally, previous research (Hohman et al. 2011; Jonker et al. 1996; Shakeel and Goghari 2017; Zullo et al. 2021) has provided controversial evidence on the associations between executive functions and subjective cognitive complaints in normal aging, therefore no additional a priori hypotheses were proposed.

## Methods

### Participants

Participants taking part in the current study were selected from an original sample of 310 community-dwellers (*M*_age_ = 78.2 years, SD = 7 years). Specifically, 89 community-based older individuals (*M*_age_ = 78.6 years, SD = 6.5 years; age range = 66–95 years), 42 males and 47 females were enrolled in the study. Their recruitment was performed through personal contacts and word-of-mouth into the community. To participate in this investigation, the following inclusion criteria had to be met: (1) being community-based, that is, the participants had to live in their own home; (2) being free from neurologic disorders (e.g., Parkinson’s disease, multiple sclerosis, and stroke); (3) reporting a Mini-Mental State Examination (Folstein et al. 1975) score > 20, to include only participants with more preserved cognitive functioning.

Using the cut-off parameters provided in the Italian validation (De Beni et al. 2007) of the CFQ (Broadbent et al. 1982), participants were assigned to the group self-reporting the lowest level of cognitive mistakes (i.e., Non-Complainers) or to the group exhibiting the highest level of cognitive complaints (i.e., Complainers), respectively. There was no significant difference in the number of participants in the two groups (*χ*^2^ = 3.247, df = 1, *p* = 0.07). Similarly, male and female participants were equally distributed in the Complainer and Non-Complainer groups (*χ*^2^ = 0.281, df = 1, *p* = 0.60). Table 1 summarizes the socio-demographic characteristics of the sample and its Mini-Mental State Examination (MMSE, Folstein et al. 1975) and Vocabulary (Wechsler 1981) scores.

**Table 1:** Sociodemographic characteristics and global cognitive efficiency (i.e., MMSE and Vocabulary scores) of participants reporting fewer cognitive failures (i.e., Non-Complainer Group) and those showing the highest CFQ scores (i.e., Complainer Group)

Variable	Non-complainer group	Complainer group	χ2	*t*	df	*p*
*n*	53	36	0.281		1	0.07
Gender			2.978		1	0.08
Males	29	13				
Females	24	23				
Marital status			0.307		1	0.58
Single/widowed	19	15				
Married	34	21				
Outdoor leisure activities			0.318		1	0.57
Yes	40	29				
No	13	7				
Type of leisure activity						
None	7	13				
Socio-cultural (e.g., University of the Third Age)	24	10				
Sport	8	5				
Handicraft (e.g., knitting, crocheting, woodwork)	14	8				
Age (years)	*M* = 79.2 (SD = 6.4)	*M* = 77.7 (SD = 6.6)		1.031	87	0.30
Years of Education	*M* = 8.5 (SD = 4.6)	*M* = 7.8 (SD = 4.4)		0.725	87	0.47
Weekly hours spent for leisure activities	*M* = 14.8 (SD = 15.7)	*M* = 12.7 (SD = 12.5)		0.662	87	0.51
Vocabulary score	*M* = 33.8 (SD = 12.9)	*M* = 32.2 (SD = 13.7)		0.541	87	0.59
MMSE score	*M* = 26.5 (SD = 2.1)	*M* = 26.5 (SD = 1.8)		0.116	87	0.91

Standard deviations are reported in parentheses

As reported in Table 1, no differences were found between the Complainer group and Non-Complainer one in terms of MMSE score, vocabulary, age, formal education (i.e., years of education), and hours per week devoted to leisure activities.

### Materials

Each participant was asked to complete the following battery of tools:

The Mini-Mental State Examination (MMSE, Folstein et al. 1975) was administered to evaluate participants’ global cognitive functioning. This test encompasses items that assess distinct cognitive processes, such as spatiotemporal orientation, attention, short and long-term memory. Scores were adjusted for age and educational attainment (Magni et al. 1996). A score < 24/30 is the cut-off used to detect the occurrence of suspected signs of cognitive decline in the Italian population. A score included between 21 and 23 indicates the suspected occurrence of signs of cognitive decline and not actual cognitive impairment. Using this criterion, 8 participants (i.e., 5 Complainers and 3 Non-Complainers) were included in the sample, since, despite the occurrence of some mild symptoms of cognitive disturbances, their successive cognitive assessment led to conclude that their cognitive efficiency was normal.

The Italian version (Orsini and Laicardi 1997) of the Vocabulary Subtest included in the Wechsler Adult Intelligence Scale (WAIS) (Wechsler 1981) was used as a control measure of cognitive functioning, that is, it was administered to assess crystalized verbal intelligence. Participants had to explain the sense of a set of 35 words, that is, they had to define (i.e., retrieving lexical information from their semantic memory) the meaning of each verbal stimulus. Each response was assessed using the criteria provided in the manual of the test. That is, a score included between 0 and 2 was assigned to each definition according to the degree of correctness and exhaustiveness. In addition, after five consecutive errors or missed responses, the task was interrupted. The maximum score was 70.

The Italian version (Belacchi et al. 2008) of the Coloured Progressive Matrices by Raven (1958) is a measure of fluid intelligence, it was used to evaluate a kind of executive function, namely the nonverbal abstract reasoning implicated in visuo-spatial problem-solving that involves novel information when familiar solutions are not available (Collins and Koechlin 2012; Shakeel and Goghari 2017). This test encompasses 36 items, and the participants had to select among six alternatives the element necessary to complete a series/geometrical pattern. For each problem correctly solved 1 score was assigned (maximum total score = 36). In the current sample, the internal consistency of this test is expressed by Cronbach’s alpha = 0.88.

The Categorical Fluency test (Novelli et al. 1986) was used as a measure of executive functioning to assess the efficiency of self-monitoring, set-shifting, and inhibition processes. The participant was invited to recall as many words belonging to a certain semantic category (e.g., animals) as possible within one minute, and to avoid repetition of the same recalled word. To perform this task, the participants had to access his/her lexical knowledge, select the appropriate stimuli target, inhibit the irrelevant ones or those already recalled and finally change the semantic category after 1 min. Three distinct semantic categories were presented, that is, animals, fruits, and car names. The correct total score was computed summing the correct responses. The mean total score for people over 70 years of age is 30.18 (SD = 7.6) (Novelli et al. 1986). In the current sample, Cronbach’s alpha is 0.73.

The Phonological Fluency test (Novelli et al. 1986) assesses self-monitoring, set-shifting, and inhibition, but, unlike the previous test, in this case, the respondent had to produce as many words as possible beginning with a certain letter of the alphabet (e.g., C). Three initial letters (i.e., F, L, and P) were provided. The administration procedure and scoring method are the same as those used for the Categorical Fluency test. A total score = 25.96 (SD = 9.27) is considered on average for people ≈ 70 years of age or older (Novelli et al. 1986). In the current sample, the internal consistency of this tool is expressed by alpha = 0.91.

The Italian version (De Beni et al. 2007) of the CFQ (Broadbent et al. 1982) was used to evaluate the occurrence of cognitive and motor errors in everyday life. This metacognitive measure includes 25 items and for each statement, the respondent was invited to self-rate how often the described error (e.g., “do you forget the name of people”) occurred in his/her daily life during the past 6 months by using a 5-point Likert scale ranging from 0 (i.e., never) to 4 (i.e., very often). The sum of the scores was calculated for each participant (maximum total score = 100). Following the Italian norms (De Beni et al. 2007), a total score ≤ 22.5 indicated the occurrence of few subjective cognitive complaints. In the current sample, the internal consistency of the CFQ scale is expressed by alpha = 0.80.

The Italian version (Fava 1983) of the Center for Epidemiological Studies of Depression Scale (CES-D, Radloff 1977) was used for the self-assessment of negative mood. This inventory encompasses 20 items assessing the occurrence of depressive symptoms during the past week on a 4-point Likert scale ranging from 0, (never or rarely) to 3 (most days or every day). The total score was computed for each participant (maximum total score = 60). A score > 16 is the Italian cut-off point indicating the occurrence of significant depressive symptoms, whereas a score ≥ 23 is used to detect those cases of suspected major depression (Fava 1983). The internal consistency is high in the general population, and in the current sample, it is expressed by alpha = 0.81.

The Italian 18-item version (Picardi et al. 2012) of Carol Ryff’s multidimensional Psychological Well-Being Scale (Ryff’s and Keyes 1995) was administered to evaluate six distinct components of positive psychological functioning, namely, Purpose in Life (i.e., the idea that one's life is goal-oriented and meaningful), Self-Acceptance (i.e., positive assessment of oneself and one's past life), Environmental Mastery (i.e., the belief to be capable to effectively manage one's life and related context), Positive Relations With Others (i.e., the ability to establish and maintain high-quality relations with others), Personal Growth (i.e., the feeling of continued growth and development as a person), and Autonomy (i.e., sense of self-determination). For each statement, the respondent had to self-rate the level of agreement on a 6-point Likert scale ranging from 1 (completely disagree) to 6 (completely agree). For each subscale, the total score was computing summing the answers provided by participants. In the current sample, the coefficient alpha for this questionnaire was 0.77.

The Italian version (Anolli 2005) of the Life Orientation Test-Revised (LOT-R, Scheier et al. 1994 is a 10-item inventory that was administered to assess positive and negative attitudes. For each item, the participant had to self-rate his/her degree of agreement on a Likert scale ranging from 1 (strongly disagree) to 5 (strongly agree). Three items are positively worded, three items are negatively worded and further 4 items are filler. After reversing the scoring for the negatively worded items, the total score was computed (maxi-mum total score is 30). A score of 10 indicates a low level of optimism (i.e., 10th percentile) (Anolli 2005). In the current sample, Cronbach's alpha has been reported to be 0.72.

### Procedure

After completion of written informed consent, each participant was tested individually in a quiet room of his/her home in two experimental sessions conducted within one week. In the first session, first, the MMSE test was presented, and then if the score was > 20, the CFQ, LOT-R, CES-D, and Ryff’s PWB were proposed. In the second experimental session, participants completed Wechsler’s Vocabulary test and executive functions measures (Belacchi et al. 2008; Novelli et al. 1986). The presentation order of the questionnaires and objective cognitive tests was counterbalanced across participants, using the Latin square procedure.

To avoid the fatigue effect of the participants, the examiner read each item aloud and she wrote the answers provided by the respondent. This procedure was adopted for all the tools, except that for the MMSE (Folstein et al. 1975), since the participants had to draw some geometrical figures, write a sentence, and perform some tasks (e.g., reading a command and performing the requested action, folding a piece of paper following the instructions given by the examiner). In total, the experimental sessions lasted approximately 75 min.

### Data analysis

Statistical analyses were performed using SPSS 24.0. The 0.05 significance level was used throughout all analyses. Descriptive statistics were calculated to examine the sociodemographic characteristics of participants and to identify both Complainers and Non-Complainers. Pearson’s correlation coefficients were computed to examine the associations between the CFQ score, each mental health index (i.e., optimism, depressive signs, and psychological well-being), and the cognitive measures (i.e., verbal fluency and Raven’s Coloured Progressive Matrices), respectively. Then, five Analyses of Covariance (ANCOVAs) and multivariate analysis of covariance (MANCOVA) were performed to determine the impact of subjective cognitive failures (i.e., Complainers vs. Non-Complainers) on mental health, executive functions, and vocabulary, using the CES-D measure as the covariate. Finally, an Analysis of Variance (ANOVA) was conducted to investigate the impact of the CFQ group on self-reported depressive signs.

## Results

First, participants exhibiting more cognitive complaints reported less optimism and psychological well-being. Indeed, significant negative associations were found between CFQ and optimism (*r* = − 0.51, *p* < 0.0005), Ryff’s PWB-Autonomy (*r* = − 0.38, *p* = 0.001), Ryff’s PWB-Personal Growth (*r* = − 0.37, *p* 0.0005), Ryff’s PWB-Environmental Mastery (r = − 0.50, *p* 0.0005), Ryff’s PWB-Positive Relations With Others (*r* = − 0.241, *p* = 0.023), Ryff’s PWB- Purpose in Life (*r* = − 0.26, *p* = 0.014) and Ryff’s PWB-Self-Acceptance (r = − 0.555, *p* < 0.0005), respectively. In contrast, a significant positive relationship was found between the CFQ and CES-D scores (*r* = 0.405, *p* < 0.0005).

Moreover, participants who were less confident in the efficiency of their mind were also less optimistic about their future (M LOT-R score of Complainers = 20, SD = 2.6) than the Non**-**Complainer group (M LOT-R score = 23.7, SD = 3.6), when the effect of self-reported depression was controlled for. Indeed, an ANCOVA documented significant main effects of the CFQ group [*F*(1,86) = 14.41, *p* < 0.0005, *η*^2^ = 0.14], and CES-D score [*F*(1,86) = 11.765, *p* = 0.001, *η*^2^ = 0.12] on the LOT-R measure. Overall, Complainers reported less optimism.

In addition, Complainers also reported less psychological well-being. Indeed, when the MANCOVA was performed to investigate the effect of the CFQ group on the Ryff’s Well-Being indexes, while controlling for the impact of the CES-D score, the Multivariate tests revealed the significant effects of the CFQ group [Wilks’ *λ* = 4.436, df = 6;81, *p* = 0.001, *η*^2^ = 0.25] and of the covariate [Wilks’ *λ* = 8.216, df = 6;81, *p* < 0.0005, *η*^2^ = 0.38]. Overall, significant differences between Complainers and Non**-**Complainers were found in Ryff’s PWB-Autonomy [*F*(1,86) = 9.03, *p* = 0.003, *η*^2^ = 0.09], Ryff’s PWB- Environmental Mastery [*F*(1,86) = 13.61, *p* < 0.0005, *η*^2^ = 0.14], and Ryff’s PWB- Self-Acceptance [*F*(1,86) = 17.76, *p* < 0.0005, *η*^2^ = 0.17] subscales, respectively. As expected, Non**-**Complainer participants reported higher well-being in the above-mentioned subscales than Complainers (see Table 2). In contrast, no significant differences between the groups were found in Ryff’s PWB-Personal Growth [*F*(1,86) = 2.83, *p* = 0.096], Ryff’s PWB- Positive Relations With Others [*F*(1,86) = 0.823, *p* = 0.367], and Ryff’s PWB- Purpose in Life [*F*(1,86) = 2.75, *p* = 0.101] conditions.

**Table 2:** Means (i.e., M), Standard Deviations (i.e., SD) reported by the Non**-**Complainers and Complainers in the self-reported measure of optimism (i.e., LOT-R), Ryff’s Psychological Well-Being subscales of Autonomy (i.e., PWB-Autonomy), Environmental Mastery (i.e., PWB-Environment), Self-Acceptance (i.e., PWB-Acceptance), Personal Growth (i.e., PWB-Growth), Positive Relations With Others (i.e., PWQ-Others), Purpose in Life (i.e., PWQ-Purpose), and depressive symptoms (i.e., CES-D)

Variable	Non-complainer group		Complainer group		*p*	*η*^2^
	*M*	SD	*M*	SD		
LOT-R	23.70	3.60	20	2.60	< 0.0005	0.14
PWB-Autonomy	16.13	2.03	14.36	2.40	0.003	0.09
PWB-Environment	14.58	2.32	11.97	2.50	< 0.0005	0.14
PWB-Acceptance	15.75	2.21	12.36	3.10	< 0.0005	0.17
PWB-Growth	14.43	1.96	12.83	2.19	0.093	
PWB-Others	15.11	2.18	13.94	2.69	0.367	
PWB-Purpose	13.06	3.07	11.31	2.96	0.101	
CES-D	8.32	6.68	16.03	11.36	< 0.0005	0.16

Furthermore, Complainers also reported more depressive signs than Non-Complainers. Indeed, an ANOVA documented the significant main effect of the CFQ group [*F*(1,87) = 16.20, *p* < 0.0005, *η*^2^ = 0.16]. Table 2 illustrates the mean scores of the Complainers and Non**-**Complainers in each mental health measure.

Then a further series of Pearson’s correlational analyses revealed no significant associations between CFQ score and Raven’s Coloured Progressive Matrices (*r* = 0.117, *p* = 276), Categorical Fluency (*r* = − 0.057, *p* = 0.60) and Phonological Fluency (*r* = 0.068, *r* = 0.601) measures, respectively.

Finally, no differences between Complainers and Non-Complainers were found in terms of their cognitive efficiency when the impact of depressive signs was controlled for. Indeed, four ANCOVAs aimed at exploring the effect of the CFQ group (i.e., Non**-**Complainers vs. Complainers) on each executive function and vocabulary measure, using the CES-D score as the covariate, showed that in Raven’s Coloured Progressive Matrices condition the main effects of group [*F*(1,85) = 0.507, *p* = 0.479], and CES-D [*F*(1,85) = 0.040, *p* = 0.841] were not significant. Moreover, the significant effect of CFQ group [*F*(1,85) = 0.118, *p* = 0.732] and CES-D [*F*(1,85) = 0.133, *p* = 0.716] were not found in the Phonological Fluency condition. In addition, in the Categorical Fluency condition, the effect of CFQ was not significant [*F*(1,85) = 0.004, *p* = 0.952], whereas there was the main effect of the covariate [*F*(1,85) = 4.51, *p* = 0.037, *η*^2^ = 0.05]. Finally, the effects of CFQ group [*F*(1,86) = 0.065, *p* = 0.8], and CES-D [*F*(1,86) = 0.369, *p* = 0.545] were not statistically significant in the Vocabulary condition. Table 3 summarizes these findings.

**Table 3:** Means (i.e., M), and Standard Deviations (i.e., SD), reported by the Non**-**Complainers and Complainers in Raven’s Coloured Progressive Matrices (i.e., Raven’s CPM), Phonological Fluency and Categorical Fluency and Wechsler’s Vocabulary (i.e., Vocabulary) conditions, respectively

Variable	Non-complainer group		Complainer group		*p*
	*M*	SD	*M*	SD	
Raven’s CPM	23.62	6.1	24.92	7.9	0.479
Phonological Fluency	27.98	8.12	26.94	10	0.732
Categorical Fluency	34.42	10.27	32.47	7.71	0.952
Vocabulary	33.77	12.93	32.22	13.74	0.8

## Discussion

The present study aimed to explore the perceived mental health and executive functions of older people who self-reported very low or very high subjective cognitive complaints assessed through the CFQ (Broadbent et al. 1982). Based on the current findings, a few conclusions can be drawn. First, following Cohen (1988) and in line with the first hypothesis (Fastame et al. 2014a), medium to large correlations were found between the cognitive complaints scores (Broadbent et al. 1982) and self-reported depressive symptoms, Ryff’s and Keyes well-being (1995), and optimism (Scheier et al. 1994) indices, respectively. Therefore, older individuals who perceived their mind as less efficient (i.e., they believed that more frequent memory, perceptual, and motor slips occurred in their everyday life) also self-reported more depressive symptoms and less psychological well-being. Second, it was also found that greater levels of subjective cognitive complaints were associated with less optimism, that is, participants who reported higher CFQ scores were also less prone to express favorable expectations about their life. Third, extending the previous evidence (Fastame et al. 2021; Fastame and Penna 2014), significant differences were found between Complainers and Non-Complainers in terms of perceived metacognition and mental health. Effect size estimates suggest that these effects can be considered moderate (when *ɳ*^2^ is included between 0.006 and 0.014, see Table 2) or large (when *ɳ*^2^ is ≥ 0.014, see Table 2). Overall, compared to the Non-Complainer group, Complainers reported more depressive symptoms, cognitive failures, and less optimism and psychological well-being. Specifically, Non-Complainers expressed a positive attitude toward their qualities, seemed aware of their strengths and weaknesses, and dealt with them in their daily life. Moreover, Non-Complainers felt confident in self-regulating their behaviors, managing a wide range of daily activities, and making decisions driven by their values and beliefs, and were shown to be very optimistic (i.e., LOT-R performance ≈70°–80° percentile). In contrast, Complainers were globally unsatisfied with themselves, they did not trust their skills and personal resources (i.e., lower Ryff’s psychological well-being (PWB) Self-Acceptance score), tended to be influenced by the opinions of others in decision making (i.e., lower Ryff’s PWB Autonomy score), and reported having difficulties in managing their everyday life (i.e., lower Ryff’s PWB Environmental Mastery score). Moreover, the Complainer group reported a mean CES-D score above the critical threshold (i.e., > 16), indicating the occurrence of significant clinical signs of depression. Consistently, following the Italian normative data [34], older individuals who self-reported higher CFQ scores also displayed less optimism (i.e., LOT-R mean score ≈ 30°–40° percentile). Overall, these outcomes suggest that older individuals who reported higher CFQ scores may be at risk of losing their autonomy in the management of their everyday life, developing a mood disorder (i.e., depression), and reducing their engagement with social life, which, in turn, represents a significant menace for the quality of life.

Fourth, consistent with previous research (Shakeel and Goghari 2017), the correlational analyses showed that self-reported cognitive failures were not associated with objectively assessed executive functions. Thus, as suggested by Toplak et al. (2013), one can conclude that “performance-based and ratings of executive function assess different aspects of cognitive and behavioral functioning” (p. 137). Extending this, the inconsistency between the objective cognitive assessment and self-reported evaluation of our participants’ mental efficiency converges with the conceptualization of a tripartite structure of mind proposed by Stanovich (2009). According to this author, a set of domain-general processes (e.g., conditioning, unconscious implicit learning) operates autonomously from the higher-order control processes when stimuli are presented (i.e., autonomous mind or System 1). In addition, there is an analytic processing system (i.e., System 2) that is organized along two-level of processing, the algorithmic level and the reflective one. The algorithmic mind encompasses cognitive processes such as fluid intelligence that are evaluated through performance-based tasks, whereas beliefs on one’s mental functioning are expressed at the level of the reflective mind. In summary, the lack of associations between the efficiency of executive functions and self-reported cognitive failure of the participants in the study would reflect the functional independence of the reflective mind from the algorithmic mind.

In addition, in the current study, a series of ANCOVAs documented no differences between Complainers and Non-Complainers in terms of cognitive functioning. Indeed, crystallized intelligence (i.e., vocabulary), verbal fluency, and non-verbal reasoning abilities were preserved both in Complainers and Non-Complainers (e.g., performance in Raven’s Coloured Progressive Matrices ≈ 50° percentile for Complainers and ≈ 75° percentile for Non-Complainers). Therefore, following Stern (2002), it is plausible to hypothesize that both Complainers and Non-Complainers used their cognitive reserve resources (i.e., adequate vocabulary and being regularly engaged in leisurely activities) to counteract the detrimental effect of aging on their cognitive functioning. In this regard, in a review, Cheng (2016) argued that physical activity is effective in slowing age-related decline in the gray matter volume of older individuals with and without cognitive impairment, and regular physical exercise seems to positively impact executive functions in late adulthood. Furthermore, the author stated that cognitively stimulating leisure activities protect against the risk for dementia (i.e., engaging different cognitive skills, including executive functions, in carrying out a wide range of tasks), and delay symptom onset (Cheng 2016). In the current sample, participants were engaged in different types of hobbies, which could have had a beneficial effect on cognitive functioning and in the empowerment of cognitive reserve. Despite this, in the present study, participants who exhibited more subjective cognitive failures tended to underestimate their actual cognitive efficiency and reported poorer perceived mental health. Therefore, following O'Connor et al. (1990), it is plausible to hypothesize that older people with significant depressive signs tended to exaggerate their perceived cognitive functions deficiencies, complaining about dealing with their everyday problems and feeling less competent in managing their lives (e.g., in the current study, Complainers reported lower scores in the Ryff’s PWB Environmental Mastery subscale).

Assuming an applied perspective, these outcomes suggest that the objective evaluation of cognitive functioning (e.g., passive and active working memory, inhibition, updating, verbal and non-verbal fluency) in late adulthood should be accompanied by the self-reported assessment of cognitive processes, as subjective cognitive concerns may be predictive of non-normative cognitive decline (Sperling et al. 2011). In addition, the concurrent evaluation of cognitive and metacognitive resources in late adulthood will allow assessing the consistency between the examinee’s residual cognitive resources and his/her level of awareness of his/her cognitive efficiency. This implies that when older individuals underestimate their actual cognitive functioning, it is crucial to inform them about the inconsistency between what they report on their perceptions of mental functioning and their objective cognitive ability, to help them to increase their self-efficacy and to decrease the occurrence of depressive symptoms. In this regard, a study showed the effectiveness of a psychoeducational intervention (i.e., consisting of eight sessions designed to inform about cognitive aging, memory beliefs, negative aging stereotypes, and compensatory metacognitive strategies to be used in everyday life) in reducing negative reactions toward the age-related cognitive decline in a group of healthy older women exhibiting cognitive concerns (Hoogenhout et al. 2012).

Moreover, these results also suggest the need to implement targeted interventions designed to enhance metacognitive and cognitive functioning in late adulthood. In this regard, there is evidence of the effectiveness of combined cognitive and metacognitive psychoeducational interventions that improve everyday functioning and psychological well-being (e.g., greater life satisfaction) and increase metacognitive knowledge (e.g., restructuring memory beliefs and increasing awareness about memory routines and their utility in the daily life) of community-based older individuals (Cheng 2016; Pearman et al. 2020).

However, some limitations of this investigation should be acknowledged. The small sample size and battery of tools used to assess the efficiency of executive functions and subjective cognitive complaints, the lack of longitudinal data to examine developmental trends in participants’ metacognitive and cognitive functioning, and the exclusive enrollment of community-dwellers, suggest caution in generalizing the current results, especially to older individuals living in nursing homes. Thus, future research should replicate the current study considering and overcoming the aforementioned limitations.

## Data Availability

The data that support the findings of this study are not publicly available due to privacy or ethical restrictions.
